# Endothelial hyperplasia: an important indicator of actual angiogenesis

**DOI:** 10.1038/sj.bjc.6600055

**Published:** 2002-02-12

**Authors:** J T Beranek

**Affiliations:** 4101 South Wappel Drive, Columbia, Missouri, MO 65203, USA

## Abstract

*British Journal of Cancer* (2002) **86**, 658. DOI: 10.1038/sj/bjc/6600055 www.bjcancer.com

© 2002 Cancer Research UK

## Sir

With a research interest in angiogenesis and antiangiogenesis (Beranek, 1988), I have read the article by Yudoh *et al* (2001) concerning microvessel density (MVD) and the concentration of vascular endothelial growth factor (VEGF) in soft tissue sarcomas. The authors have found that no significant correlation exists between MVD and the concentration of VEGF in the tumours and that increased VEGF concentration in tumours and not MVD is a negative prognostic factor for the disease outcome. This is an unexpected result deserving further discussion because VEGF is of central importance in practically all cases of physiologic and pathologic angiogenesis (Senger, 2001).

Yudoh *et al* (2001) have determined MVD by the immunopositivity of endothelial cells for Factor VIII-related antigen. In their Figure 1B, numerous blood vessels are seen. A majority of them manifest a feature which the original authors overlooked: their walls are thick and manifest the immunopositivity which is not limited to their most internal cells but extends across the vessel wall, implying that these walls are lined by more than one layer of endothelial cells. This phenomenon may be explained by a dedifferentiation of endothelial cells induced by angiogenesis (Beranek, 1988). Dedifferentiated endothelial cells, having lost their polarity and contact inhibition, are able to form hyperplastic capillary sprouts composed of several layers of endothelial cells (Beranek, 1988). These sprouts have the capacity to develop into different vascular structures: (a) into more or less ectatic capillaries lined by one layer of endothelial cells; (b) into hyperplastic capillaries lined by more than one layer of endothelial cells (Yudoh, *et al* 2001, Figure 1B); (c) into muscle vessels by the transdifferentiation of peripheral undifferentiated endothelial cells into smooth muscle cells (Beranek, 1998), and (d) into endothelial glomeruloid formations by the death of some and the redifferentiation of other undifferentiated endothelial cells (Beranek, 1991; Figure 1).

Recently, Sundberg (2001) injected adenoviral vectors expressing murine VEGF intradermally into mouse ears and provoked a formation of endothelial glomeruloid bodies, confirming thereby experimentally the hypothesis that vascular endothelial cells dedifferentiate during angiogenesis and are able to form hyperplastic vascular structures lined by more than one layer of endothelial cells. In order to quantify angiogenesis one must, therefore, take into consideration not only MVD but also the endothelial hyperplasia of newly formed vessels (Beranek, 2001). In this case, it is quite probable that VEGF tumour concentration will correlate with morphologic manifestations of tumour angiogenesis and that both these factors will be implicated in the disease prognosis. Yudoh *et al* (2001) divided their patients in groups with or without local recurrence and with or without metastasis. It would be interesting to know whether the groups without local recurrence and without metastasis manifest less endothelial hyperplasia than their counterparts.
